# Patient-Reported Outcomes of Digital Versus Conventional Impressions for Implant-Supported Fixed Dental Prostheses: A Systematic Review and Meta-Analysis

**DOI:** 10.3390/jpm15090427

**Published:** 2025-09-05

**Authors:** Aspasia Pachiou, Evangelia Zervou, Nikitas Sykaras, Dimitrios Tortopidis, Alexis Ioannidis, Ronald E. Jung, Franz J. Strauss, Stefanos Kourtis

**Affiliations:** 1 Clinic of Reconstructive Dentistry, Center for Dental Medicine, University of Zurich, Plattenstrasse 11, CH-8032 Zurich, Switzerland; aspapachiou@dent.uoa.gr (A.P.); alexis.ioannidis@zzm.uzh.ch (A.I.); ronald.jung@zzm.uzh.ch (R.E.J.); franz.strauss@zzm.uzh.ch (F.J.S.); 2 Independent Researcher, Loutraki, 20300 Corinth, Greece; ezervou@dent.uoa.gr; 3 Department of Prosthodontics, School of Dentistry, National Kapodistrian University of Athens, Thivon 2, 11527 Athens, Greece; nsykaras@dent.uoa.gr; 4 Department of Prosthodontics, School of Dentistry, Aristotle University of Thessaloniki, University Campus, 54124 Thessaloniki, Greece; dtortopi@dent.auth.gr; 5 Center for Studies and Innovation in Dentistry, Faculty of Dentistry, Universidad Finis Terrae, Santiago 7501016, Chile

**Keywords:** digital workflow, digital impression, intraoral scanning, patient perception, PROM, patient-reported outcome, implant impression, precision dentistry, personalized medicine

## Abstract

**Background/Objectives:** To compare patient-reported outcome measures (PROMs) between digital and conventional impression techniques for implant-supported fixed dental prostheses (iFDPs). **Methods:** A systematic literature search was conducted in PubMed, Embase, Scopus, and the Cochrane Library databases up to June 2025, following PRISMA guidelines. Human clinical studies reporting PROMs between digital and conventional impression techniques for iFDPs were included. Studies using structured, but not necessarily validated, questionnaires were eligible. Two reviewers independently performed study selection, data extraction, and risk of bias assessment. Where possible, meta-analyses were conducted using a random-effects model to pool comparable outcomes across studies using mean differences (MD) or standardized mean differences (SMD) with 95% confidence intervals (CIs). **Results:** Out of 1784 records screened, eighteen studies were included. Most studies showed that digital impressions were associated with higher patient satisfaction, compared to conventional impressions. Ten studies contributed data to at least one outcome; pooled analyses included the following: overall satisfaction (k = 5), comfort (k = 7), gagging/nausea (k = 5), esthetic satisfaction (k = 2), unpleasant taste (k = 5), anxiety (k = 5), discomfort (k = 2), pain (k = 5), and overall discomfort (k = 5). Digital impressions were significantly favored (*p* < 0.05) for anxiety (MD = 13.3, 95% CI: −22 to −4.5), nausea (MD = −26.4, 95% CI −46.8 to −6.0), bad taste (MD = −34.8, 95% CI −58.3 to −11.3), discomfort (SMD = −2.24, 95% CI −3.51 to −0.98), comfort (SMD = 1.77, 95% CI: 0.60 to 2.94), perceived procedure time (SMD = 0.96; 95% CI 0.29 to 1.62), and overall satisfaction (SMD = 0.55; 95% CI 0.01 to 1.09). No statistically significant differences were found for pain or esthetic evaluation. Substantial between-study heterogeneity was observed among the included studies. **Conclusions:** Current evidence indicates that digital impression workflows enhance the overall patient experience for implant-supported fixed restorations, especially in domains linked to comfort and procedural efficiency. These findings support PROM-informed personalization of impression workflows: screening for gagging, anxiety, or intolerance to impression materials could guide patient-tailored use of intraoral scanning while acknowledging no consistent advantage for pain or esthetic perception.

## 1. Introduction

Accurate impressions are fundamental to the long-term success of implant-supported prostheses because they replicate intraoral structures and influence the fit and function of the definitive restoration [1,2]. Conventional techniques, typically using polyether or polyvinylsiloxane [3], have long been considered the clinical benchmark. However, they are associated with drawbacks such as gag reflex induction, patient anxiety, and material-related inaccuracies [4]. These material-related accuracies are particularly relevant in implant prosthodontics. Even minor discrepancies at the micron level may compromise peri-implant health with consequent biofilm accumulation and patient comfort. Unlike tooth-borne restorations, implants lack a periodontal ligament and therefore accommodate inaccuracies poorly [4].

Digital intraoral scanning has emerged as a contemporary alternative [5,6]. By generating a direct three-dimensional dataset, intraoral scanners (IOS) eliminate tray materials, reduce chairside time, and provide real-time feedback that allows for immediate correction of captured errors [4]. Several studies report patients’ preferences for digital impressions, citing less discomfort, a diminished gag reflex, and lower procedural anxiety [7,8,9]. These advantages may be particularly valuable in implant therapy, where patient experience can significantly influence treatment acceptance and overall satisfaction [10,11,12].

From a technical standpoint, digital impressions achieve marginal fits that are comparable to and, in many single-unit cases, superior to those produced by conventional methods, largely due to the elimination of model fabrication steps [12,13,14]. Nevertheless, challenges remain when scanning full arches or multiple implants, especially in posterior regions or on reflective abutment surfaces [15]. Some studies suggest that despite improved comfort, these benefits may not consistently translate into better esthetic or functional satisfaction once the prosthesis is delivered [16]. In addition, digital workflows have not been adopted everywhere yet, partly because they require time to learn and can be expensive to set up [17].

Given the focus on patient-centered care and the elective, esthetic nature of many implant cases, clarifying whether digital impressions actually improve patient-reported outcomes (PROs) is important. A patient-reported outcome measure (PROM) is a questionnaire designed to assess a patient-reported outcome (PRO) [18,19,20,21]. PROMs can capture patients’ own views of their oral health and its day-to-day impact. Incorporating these insights into care planning increases patient involvement in decisions, often leading to increased satisfaction and improved clinical outcomes [22,23,24,25,26].

Although existing systematic reviews highlight the clinical efficiency and convenience of digital impressions [9,12,27,28,29,30], evidence on their impact on PROMs remains fragmented. Notably, implant-specific PROMs, including comfort, anxiety, willingness to repeat the procedure, and overall satisfaction, are inconsistently defined and are often treated as secondary outcomes. Furthermore, studies use a variety of questionnaires, many of which are not validated for implant populations, further limiting comparability. These issues limit the ability to draw firm conclusions about patient benefit in implant prosthodontics. Building on prior work, this review (i) performs a quantitative meta-analysis with pooled estimates and formal heterogeneity assessment; (ii) evaluates nine PROM domains for a more granular appraisal of patient experience; and (iii) provides an up-to-date synthesis of this rapidly evolving area of intraoral scanners and clinical workflows.

Precision medicine aims to tailor interventions to the characteristics, preferences, and risks of individual patients. In dentistry, framing PROMs as actionable inputs for workflow selection positions digital impressions as a personalized strategy for patients likely to experience discomfort with elastomeric materials while recognizing that esthetic outcomes may not differ once definitive prostheses are delivered. PROMs function as patient-centered “phenotypic” markers that meaningfully influence acceptance and adherence.

The present review, therefore, aimed to compare current evidence on PROMs between digital and conventional impressions for implant-supported fixed restorations. The null hypothesis stated that digital impressions for iFDPs would not result in improved PROMs compared to the conventional method.

## 2. Materials and Methods

This systematic review was conducted following the Preferred Reporting Items for Systematic Reviews and Meta-Analyses (PRISMA) guidelines to ensure methodological rigor and transparency [31]. The research question was structured using the PICO (Population, Intervention, Comparison, Outcome) framework: “In patients receiving implant-supported fixed restorations (P), do digital impressions (I) compared to conventional impressions (C) result in improved patient-reported outcomes? (O)”. This review was registered in the PROSPERO database (PROSPERO ID: CRD42024607287).

### 2.1. Information Sources and Search Strategy

An extensive search strategy was developed based on the PICO framework. Electronic searches were conducted across multiple databases, including PubMed, Embase, Scopus, and the Cochrane Library. Additionally, reference lists of relevant reviews and included primary studies were screened to identify further eligible articles. A systematic search of the gray literature (OpenGrey) was also performed along with a review of registered but unpublished trials in ClinicalTrial.gov as well.

The search was completed on 16 June 2025, with no date restrictions applied to study inclusion. Search strategies were tailored to each database using controlled vocabulary (MeSH/Emtree) and equivalent free-text synonyms, combining implant terms (P), impression workflow terms (I), and patient-reported outcomes (O) with Boolean operators; a concise summary appears in Table 1, and full strings are provided in Supplementary Table S1.

### 2.2. Eligibility Criteria

The inclusion criteria for this review were as follows:

- Any type of clinical study, including randomized controlled trials (RCTs), cross-sectional studies, cohort studies, and case–control studies.
- Studies with more than five participants who received one or more implants restored using either digital or conventional protocols.
- Articles published in English, specifically comparing digital and conventional implant impressions and reporting on patient perception, comfort, anxiety, or nausea during the impression-taking process.

Exclusion criteria included the following:

- Case reports, case series, expert opinions, preclinical studies, and studies lacking data on impression materials, procedures, or intraoral scanners.
- Unpublished reports, abstracts, or papers that did not address both conventional and digital impression techniques.
- Studies lacking sufficient data for analysis, including those based solely on surveys or retrospective chart reviews, or those that did not allow for the extraction of relevant outcome measures.

The ≥5 threshold was selected to minimize small-study bias and imprecise variance from case reports/very small series while preserving inclusiveness for early implant PROM evidence. Studies with mixed tooth- and implant-borne cohorts were included only when implant-specific data were extractable.

### 2.3. Study Selection

The study selection process comprised three sequential stages. Prior to screening, the two independent reviewers completed a pilot calibration on a random sample of 50 records to harmonize the application of eligibility criteria. Subsequently, titles and abstracts were screened independently by two investigators (A.P. and E.Z.). Any disagreements were resolved by discussion or adjudication by a third reviewer (S.K.). In the final stage, full texts of all potentially eligible studies were meticulously assessed for eligibility based on the predefined inclusion and exclusion criteria. Any discrepancies were resolved through discussion among all three reviewers (A.P., E.Z., and S.K.). Inter-rater agreement was quantified using Cohen’s κ at both stages. Studies that met the eligibility criteria were included in the final synthesis. A final review of all included articles was performed jointly by the three investigators to ensure accuracy and consistency.

Records retrieved from all databases were imported into Rayyan (Qatar Computing Research Institute, Doha, Qatar) for management and screening. The results were detected for duplicates and de-duplicated within the platform. Duplicates were automatically identified and manually deduplicated prior to screening.

### 2.4. Data Extraction and Method of Analysis

Data extraction was performed independently by two reviewers (AP, EZ). To ensure accuracy, all extracted data was cross-checked, and any discrepancies were resolved through discussion until consensus was achieved. For each included study, the following information was collected: authors’ names, year of publication, study design, type of restoration, implant system used, restoration location, number of participants, impression material, type of intraoral scanner used, tools used to assess patient preference, outcome measures, and main conclusions.

### 2.5. Risk of Bias (Quality Assessment)

The quality of each study was appraised using design-specific instruments. Randomized clinical trials (RCTs) were evaluated with the Cochrane RoB 2; non-randomized interventional studies with the JBI quasi-experimental checklist; observational cohort and cross-sectional studies with the NIH cohort/cross-section tool; and before–after studies with the NIH before–after checklist. Two reviewers (A.P., E.Z.) independently rated each article, with any disagreements resolved by a third author (F.J.S.). Ratings for individual domains were synthesized into an overall risk of bias (low, moderate/some concerns, or high risk of bias).

### 2.6. Statistical Analysis

Mean scores of PROMs were pooled across studies. When outcomes were measured using the same scale, the results were expressed as mean differences (MD) with 95% confidence intervals. For outcomes measured with different scales, scores were standardized and presented as standardized mean differences (SMD) to enable comparison. All outcomes were synthesized with a random-effects model to account for variability between study settings. Cross-over trials were synthesized using paired differences with SEs from the paired SD. Parallel-arm trials were synthesized by using standard calculations from group means and SDs under a random-effects model.

Heterogeneity was estimated using restricted maximum likelihood and summarized with I^2^, interpreted as low (0–40%), moderate (30–60%), substantial (50–90%), or considerable (≥75%). To reduce small-sample bias and account for unequal study sizes, Hedges’ g was also reported. All analyses were performed in R Studio version 2024.12.1 using the “meta” and “metafor” packages.

## 3. Results

### 3.1. Search and Selection

**Figure 1:**
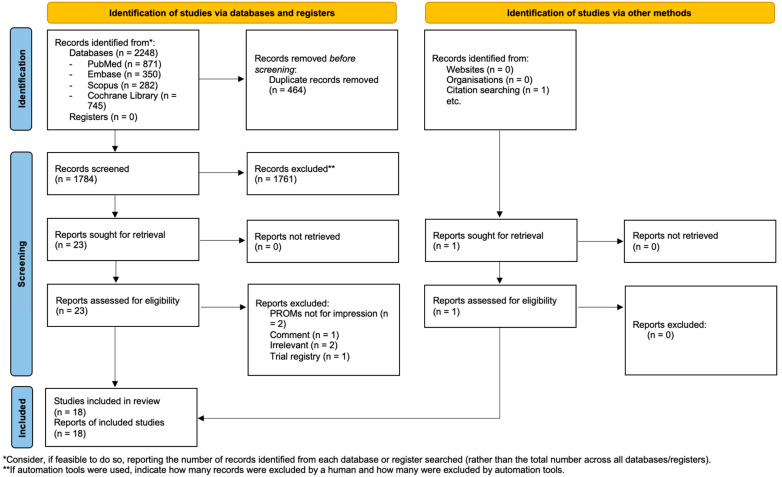
PRISMA flow diagram.

A total of 1784 studies were identified through the initial database search on PubMed, Embase, Scopus, and the Cochrane Library. Following PRISMA guidelines, all abstracts were systematically screened. After removing duplicates and applying the inclusion criteria, 23 studies were selected for full-text review. Of these, six studies were excluded for not meeting the eligibility criteria (PRISMA flow diagram, Figure 1). Finally, 17 studies met the inclusion criteria. An additional study was identified through cross-referencing, bringing the total number of included studies to 18. Inter-rater agreement was substantial (Cohen’s κ = 0.93 at the abstract level and 1 at the full-text level). The study selection process is illustrated in the PRISMA flow diagram (Figure 1) [32].

### 3.2. Study Characteristics

The main table provides an overview of the characteristics of the studies included in this review (Supplementary Table S1). All studies were published between 2015 and 2024 and were classified as clinical studies. Among these, five (27.78%) were crossover RCTs [33,34,35,36,37] and four (22.22%) were parallel-arm RCTs [14,16,38,39]. Another four (22.22%) studies were non-randomized clinical studies [40,41,42,43]. Two studies (11.11%) were retrospective clinical studies [44,45], and two (11.11%) were cross-sectional [46,47]. One additional study (5.6%) was a self-controlled non-randomized study [48].

In terms of the types of restorations studied, thirteen studies (72.22%) focused on screw-retained single crowns. Two studies (11.11%) examined full-arch implant-supported restorations, one study investigated about 3-unit fixed partial dentures (FDPs), and the remaining two studies (11.11%) reported on single crowns and FDPs.

Regarding the locations of the implant restorations, the majority of studies (77.78%) focused on posterior sites. Two studies (11.11%) addressed full arches, one study (5.5%) included both anterior and posterior sites, and one study focused specifically on full-arch mandibular impressions (7.6%). Only one study (7.6%) did not specify the location of the implant impressions.

All selected studies provided detailed information on the impression materials used in conventional impressions. Ten studies (55.56%) used polyether, while the remaining eight studies (44.44%) utilized polyvinylsiloxane.

### 3.3. Risk of Bias

Among the 18 included studies, three (16.7%) were rated low risk of bias, thirteen (72.2%) moderate/some concerns, and two (11.1%) high risk. Summarized ratings are provided in Supplementary Table S2. The PRISMA checklist is provided in Supplementary Table S3.

### 3.4. Pooled Analysis

Nine studies reported on patient comfort when comparing digital with conventional impressions for implant-supported fixed restorations. Of these, seven used questionnaires with the same directional scale, while two used reverse scales (where lower scores indicated greater comfort) and were analyzed separately.

**Figure 2:**
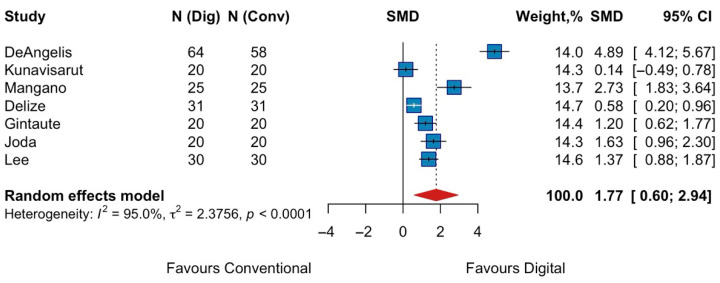
Forest plots of pooled patient comfort comparing digital versus conventional implant impression. Diamond widths and horizontal bars show 95% CIs, and box sizes indicate relative weight in the meta-analysis. The boxes and pooled estimate (diamond) resulted from the random-effects-restricted maximum likelihood approach. SMD = standardized mean difference.

A meta-analysis was conducted using the standardized mean differences (SMD) from the seven studies with consistent direction. The pooled effect size was 1.77 (95% CI: 0.60 to 2.94) with a statistically significant overall effect (z = 2.97 (*p* = 0.003)), suggesting that intraoral scanning was associated with greater patient comfort compared to conventional impressions. Heterogeneity was high (I^2^ = 95%, Q-test = 119.50, *p* < 0.0001) (Figure 2), suggesting substantial variability across study results.

**Figure 3:**
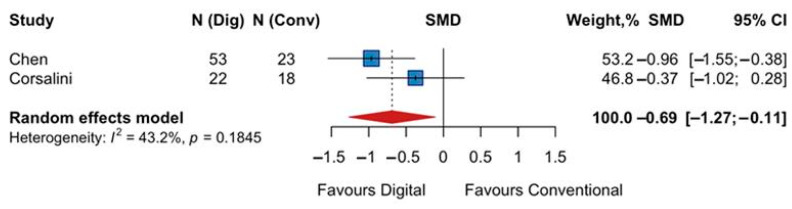
Forest plot of pooled patient comfort comparing digital versus conventional implant impression using a reverse scale (lower scores indicate more comfort). Diamond widths and horizontal bars show 95% CIs, and box sizes indicate relative weight in the meta-analysis. The boxes and pooled estimate (diamond) resulted from the random-effects-restricted maximum likelihood approach. SMD = standardized mean difference.

A separate analysis was conducted for the two trials that employed a reverse comfort scale, where 0 indicated the greatest comfort. This analysis also significantly favored the digital workflow, with a pooled SMD of −0.69 (95% CI −1.27 to −0.11; z = −2.33; *p* = 0.02). Since lower scores represented more comfort in these studies, the negative effect estimate again indicates superior patient experience with digital impressions. Heterogeneity was moderate (I^2^ = 43%) (Figure 3).

**Figure 4:**
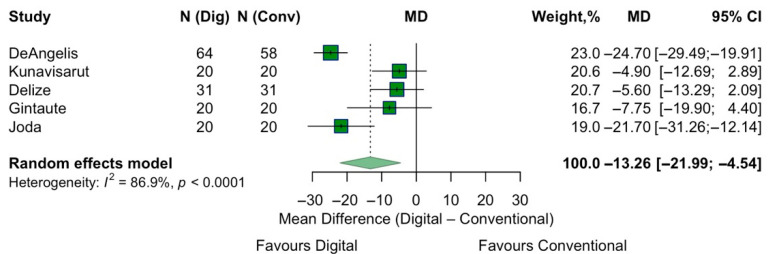
Forest plot of pooled patient anxiety comparing digital versus conventional implant impression. Diamond widths and horizontal bars show 95% CIs, and box sizes indicate relative weight in the meta-analysis. The boxes and pooled estimate (diamond) resulted from the random-effects-restricted maximum likelihood approach. MD = mean difference.

Anxiety levels associated with the two procedures were assessed in five studies, all using the same VAS scale. The pooled estimate showed an overall MD of −13.3 (95% CI: −22 to −4.5) in favor of the digital impressions, suggesting significantly lower anxiety with digital impressions. The overall effect was statistically significant (z = −2.98, *p* = 0.003). These findings suggest that patients experience less anxiety with digital implant impressions compared to conventional ones. Heterogeneity was considerable (I^2^ = 86.9%) (Figure 4).

**Figure 5:**
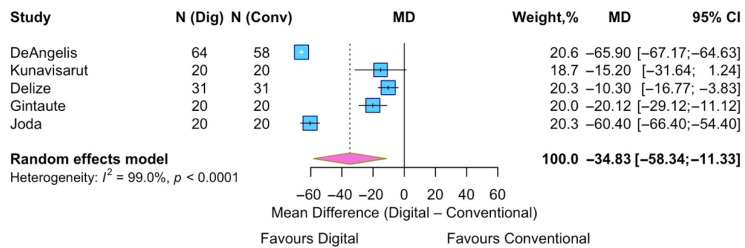
Forest plot of pooled patient perception of unpleasant taste comparing digital versus conventional implant impression. Diamond widths and horizontal bars show 95% CIs, and box sizes indicate relative weight in the meta-analysis. The boxes and pooled estimate (diamond) resulted from the random-effects-restricted maximum likelihood approach. MD = mean difference.

Five studies were included in the analysis of patients’ perception of unpleasant taste. The pooled analysis indicated that digital impressions were associated with a significantly reduced perception of unpleasant taste relative to conventional techniques, with a pooled MD = −34.8 (95% CI −58.3 to −11.3; effect z = −2.98 (*p* = 0.004)). Considerable heterogeneity was found (I^2^ = 99%, *p* < 0.0001) (Figure 5).

**Figure 6:**
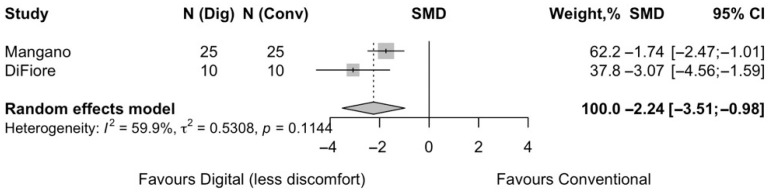
Forest plot of pooled patient discomfort comparing digital versus conventional implant impression. Diamond widths and horizontal bars show 95% CIs, and box sizes indicate relative weight in the meta-analysis. The boxes and pooled estimate (diamond) resulted from the random-effects-restricted maximum likelihood approach. SMD = standardized mean difference.

Two studies reported on patient discomfort when impressions are taken digitally rather than conventionally. The pooled synthesis indicated a statistically significant reduction in patient-reported discomfort in cases of digital impressions with a pooled effect of SMD = −2.24 (95% CI −3.51 to −0.98; z = −3.48 (*p* = 0.0005). Moderate heterogeneity was detected (I^2^ = 60%) (Figure 6).

**Figure 7:**
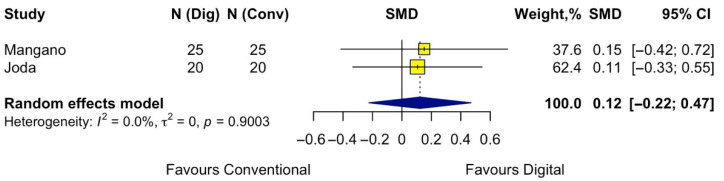
Forest plot of pooled patient-reported esthetic satisfaction comparing digital versus conventional implant impression. Diamond widths and horizontal bars show 95% CIs, and box sizes indicate relative weight in the meta-analysis. The boxes and pooled estimate (diamond) resulted from the random-effects-restricted maximum likelihood approach. SMD = standardized mean difference.

Concerning esthetic satisfaction, two studies were included in the meta-analysis. The pooled analysis did not indicate a statistically significant difference in patient-reported esthetic satisfaction between digital and conventional impressions (pooled effect SMD = 0.12; 95% CI −0.22 to 0.47; *p* = 0.49). Low heterogeneity was detected (I^2^ = 0%) (Figure 7).

**Figure 8:**
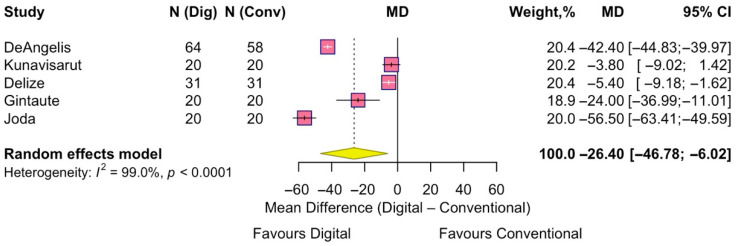
Forest plot of pooled patient-reported nausea comparing digital versus conventional implant impression. Diamond widths and horizontal bars show 95% CIs, and box sizes indicate relative weight in the meta-analysis. The boxes and pooled estimate (diamond) resulted from the random-effects-restricted maximum likelihood approach. MD = mean difference.

Five studies were included in the analysis of nausea. The pooled analysis showed that digital impressions were associated with significantly reduced nausea compared to conventional techniques, with a pooled MD = −26.4 (95% CI −46.8 to −6.0; effect z = −2.54, *p* = 0.01). Considerable heterogeneity was detected (I^2^ = 99%) (Figure 8).

**Figure 9:**
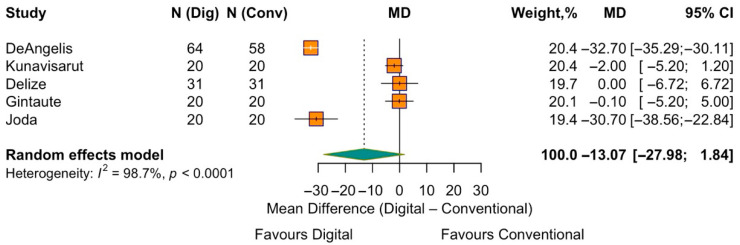
Forest plot of pooled procedure-related pain comparing digital versus conventional implant impression. Diamond widths and horizontal bars show 95% CIs, and box sizes indicate relative weight in the meta-analysis. The boxes and pooled estimate (diamond) resulted from the random-effects-restricted maximum likelihood approach. MD = mean difference.

Procedure-related pain was assessed in five studies, all using the same VAS scale. The random-effects model did not show a statistically significant difference for the two impression methods (pooled MD = −13.1, 95% CI −28.0 to 1.8; *p* = 0.086). The between-study heterogeneity was high (I^2^ = 98.7%) (Figure 9).

**Figure 10:**
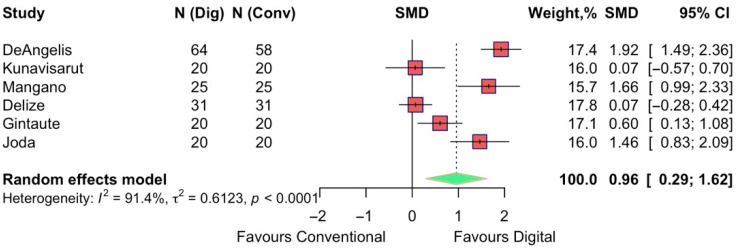
Forest plot of pooled patient-reported time perception comparing digital versus conventional implant impression. Diamond widths and horizontal bars show 95% CIs, and box sizes indicate relative weight in the meta-analysis. The boxes and pooled estimate (diamond) resulted from the random-effects-restricted maximum likelihood approach. SMD = standardized mean difference.

The patient-reported time perception associated with the two procedures was assessed in six studies. The pooled estimate indicated an overall SMD = 0.96 (95% CI 0.29 to 1.62) in favor of the digital impressions, suggesting that patients considered digital impressions to be faster. The test for overall effect was statistically significant (z = 2.82, *p* = 0.005). Heterogeneity was considerable (I^2^ = 91.4%) (Figure 10).

**Figure 11:**
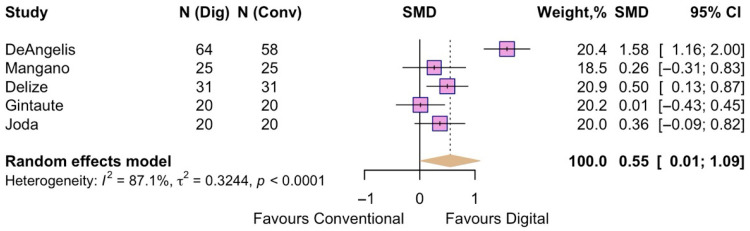
Forest plot of pooled overall patient satisfaction comparing digital and conventional implant impressions. Diamond widths and horizontal bars show 95% CIs, and box sizes indicate relative weight in the meta-analysis. The boxes and pooled estimate (diamond) resulted from the random-effects-restricted maximum likelihood approach. SMD = standardized mean difference.

The overall patient satisfaction was evaluated in five studies. The pooled effect size was SMD = 0.55 (95% CI 0.01 to 1.09) with a statistically significant overall effect (z = 2.01, *p* = 0.045), suggesting that intraoral scanning is associated with increased overall patient satisfaction compared to the conventional method. Heterogeneity was considerable (I^2^ = 87.1%) (Figure 11), suggesting substantial variability across study results.

## 4. Discussion

This systematic review and meta-analysis compared digital impressions for implant-supported fixed restorations with conventional ones in terms of patient-reported outcome measures (PROMs).

Overall, the null hypothesis was partially rejected. Digital impressions were associated with significant improvements in several PROMs, including comfort, nausea, unpleasant taste, anxiety, discomfort, patient-reported time perception, and overall satisfaction. Conversely, no significant differences were detected between the two techniques regarding esthetic perception and pain, partially supporting the null hypothesis for those outcomes.

Comfort is important during the impression procedure, which involves physical ease, the ability to breathe freely, and psychological relaxation. The present meta-analysis showed a significant improvement in patient comfort with digital impressions and a corresponding reduction in discomfort. These findings are consistent with other systematic reviews [12,29]. The enhanced comfort may be explained by the absence of bulky trays or viscous elastomer materials that contact the palate or oropharynx [33], reduced sensation of breathlessness [37,46], and the possibility to pause the scanning procedure when needed [10,16]. This ergonomic advantage of IOS does not, per se, reduce accuracy, provided that acquisition is resumed with sufficient overlap and artifacts are rescanned according to manufacturer guidance. Comfort also influences treatment decisions, as patients increasingly prefer clinicians who use fully digital workflows over those who still rely on conventional impression methods [47]. This has practical implications for treatment acceptance, especially in complex, multi-visit implant therapy plans.

Anxiety is a major barrier to dental care. The meta-analysis showed that digital impressions significantly reduced procedural anxiety, aligning with findings from previous studies [9,12]. Lower anxiety has tangible clinical benefits: it improves patient understanding of treatment [49], supports better oral hygiene, and increases adherence to follow-up visits [50]. Qualitative data further reinforce this: digital scanning was perceived as less distressing, with fewer feelings of helplessness and less fear of needing impression repetition [42]. Scanner-based workflows also reduced somatic anxiety symptoms, such as shortness of breath [37].

Unpleasant taste, frequently linked to conventional materials, was also significantly reduced with digital impressions. Elastomeric materials may leach residual monomers or flavoring agents described as bitter or chemical. Digital workflows eliminate these substances entirely. This finding aligns with prior reviews [9,30] and qualitative data that associate bad taste with cold-induced hypersensitivity [46] and the spread of materials across the palate [33]. Digital impressions avoid both chemical and mechanical triggers, leading to a more pleasant experience.

Nausea and gagging, often caused by material bulk and contact with the soft palate, were significantly less frequent with digital impressions. These results are supported by other meta-analyses [9,30] and qualitative studies [14,46]. Since gag reflexes are closely linked to dental anxiety [51], the nausea-reducing effects of digital scanning may further enhance cooperation by addressing multiple sources of distress simultaneously.

Pain, though an important concern, showed no significant differences between the two methods in this review, consistent with findings by de Paris Matos et al. (2023) [30]. Notably, some discomfort has been reported with digital scanners due to the size of the scanner head [46], although this likely varies by device model and design. Qualitative data, however, still suggest a generally less painful experience with digital impressions [40].

Esthetic satisfaction did not differ significantly between impression techniques. Both workflows ultimately feed into similar CAD/CAM production processes, which may explain the comparable outcomes. Several studies, including long-term trials, support this equivalence [34,35,52].

Time perception was also improved with digital impressions. Across included studies, patients consistently reported a shorter perceived duration with intraoral scanning compared with conventional impressions. While early studies using first-generation scanners did not detect a difference [33], newer technologies have improved speed and efficiency, aligning subjective impressions with objective time savings [36]. Objective chairside time measurements also indicate a time advantage for intraoral scanning [29].

Overall satisfaction, which reflects a combination of comfort, taste, anxiety, efficiency, and more, was significantly higher with digital impressions. Qualitative findings from additional studies not included in the meta-analysis further support this result [33,40,42,45]. Recent clinical trials also report that most patients would choose digital impressions again and recommend them to others [14]. Even earlier studies using slower scanners documented high satisfaction levels [33]. In addition, digital impressions are less prone to distortion due to the absence of impression materials, contributing to accurate restorations and improved patient satisfaction [53,54].

Consistent improvements in comfort, anxiety, nausea, unpleasant taste, perceived procedure time, and overall satisfaction with digital impressions support PROM-guided personalization. A brief preprocedural screen (e.g., VAS for gagging/anxiety and a material-tolerance item) can direct scanning to patients most likely to benefit, whereas others may proceed with either workflows based on logistics or cost. Because pain and esthetic perception show no consistent differences, personalization should emphasize intolerance-related domains and procedural efficiency within shared decision-making. Looking ahead, AI leveraging e-health data may enhance diagnosis and planning, enabling individualized reconstructive workflows and skill-based personalized curricula [55,56].

Strengths of this systematic review include the implant-only scope, PROMs specified as primary endpoints, quantitative synthesis across nine domains of PROMs, and a recent multi-database search. However, several limitations should be acknowledged. Many included studies had a high or unclear risk of bias, and heterogeneity in outcome measures reduced the confidence in pooled estimates. The substantial between-study heterogeneity observed across several PROM domains is compatible with patient-level variability (for instance, propensity for gagging/anxiety), underscoring the rationale for personalized, PROM-guided workflow selection rather than a uniform approach. Confounding factors, such as operator experience, patient expectations, and variations in clinical protocols, may have influenced the results. Moreover, real-world adoption of digital impressions is limited by high initial costs, the need for clinician training, and technological integration challenges. Finally, the search was not formally validated against a sentinel set; however, multi-database coverage, citation chasing, reference screening, “snowballing”, and an updated run were undertaken to enhance recall, though some omissions remain possible.

## 5. Future Directions

Future high-quality, standardized randomized controlled trials are needed to validate these findings across broader populations and settings. Longitudinal studies assessing the impact of impression experience on long-term, follow-up adherence would also be valuable.

## 6. Conclusions

Within the limitations of the available evidence, digital implant impressions for iFDPs are associated with improvements in several PROMs, including comfort, nausea, unpleasant taste, anxiety, discomfort, patient-reported time perception, and overall satisfaction. These findings support PROM-informed personalization of impression workflows: screening for gagging, anxiety, or intolerance to impression materials could guide patient-tailored use of intraoral scanning while acknowledging no consistent advantage for pain or esthetic perception.

## Figures and Tables

**Table 1:** Summary of database search strategies. Full line-by-line search strings are provided in Supplementary Table S1.

Controlled Vocabulary (Examples)	Free-Text Keywords (Examples)
P (implants): Dental Implants; Dental Prostheses, Implant-Supported; Single-Tooth Dental Implants; Dental Implantation, Endosseous (MeSH). I (impressions): Dental Impression Technique; Dental Impression Materials; Computer-Aided Design (MeSH). O (PROMs): Patient Satisfaction; Patient Outcome Assessment; Nausea; Patient Preference (MeSH).	P: “dental implant*”, “implant crown*”, “implant bridge*”, “all-on-4/6”, “full arch fixed denture*”, “screw retained”, “cement retained”. I: “digital impression*”, “intraoral scan*”, “optical impression*”, “digital scan*”, “dental scanner*”, “scanbod*”, “implant impression*”. O: “patient reported outcome*”, “PROs”, “PROMs”, “patient comfort”, “preference*”, “anxiety”, “discomfort”, “taste”, “gag reflex”, “nausea”.

## Data Availability

The raw data supporting the conclusions of this article will be made available by the authors on request.

## Supplementary Materials

The following supporting information can be downloaded at https://www.mdpi.com/article/10.3390/jpm15090427/s1: File S1: Search strategies; Table S1: Main table; Table S2: Summary of the quality assessment; Table S3: PRISMA 2020 Checklist.
