# Supplementary material for: Patient-Reported Outcomes of Digital Versus Conventional Impressions for Implant-Supported Fixed Dental Prostheses: A Systematic Review and Meta-Analysis
Source: J Pers Med. 2025 Sep 5;15(9):427. doi: 10.3390/jpm15090427 (PMC12471007; doi:10.3390/jpm15090427)
Supplement: Supplementary file 1 [file jpm-15-00427-s001.zip › jpm-3799588-supplementary File S1 & Tables S1 and S2.pdf]

Supplementary File S1: Search strategies

Search strategy for PubMed:

P: ((((((((((((((((((((((((((dental implant[MeSH Terms]) OR (dental implants[MeSH Terms])) OR (dental prostheses, implant supported[MeSH Terms])) OR (dental prosthesis, implant supported[MeSH Terms])) OR (dental implants, single tooth[MeSH Terms])) OR (single tooth dental implants[MeSH Terms])) OR (dental implantation, endosseous[MeSH Terms])) OR (dental implant*)) OR (implant crown*)) OR (implant bridge*)) OR (implant FDP)) OR (implant fixed partial denture*)) OR (implant supported single unit crown*)) OR (fixed implant restoration*)) OR (fixed implant prosthesis)) OR (fixed implant prostheses)) OR ("all on four")) OR ("all on six")) OR (full arch fixed denture*)) OR (screw retained)) OR (cement retained)) OR (dental implant*)) OR (implant supported restoration*)) OR (single unit dental implant*))) OR (all-on-4)) OR (all-on-six)) OR (full arch restoration)) OR (implant restoration*)

I: ((((((((((((dental impression technique[MeSH Terms]) OR (dental impression material[MeSH Terms])) OR (dental impression materials[MeSH Terms])) OR (computer aided design[MeSH Terms]))) OR (digital impression*)) OR (dental impression*)) OR (intraoral scan*)) OR (optical impression*)) OR (digital scan*)) OR (dental scanner*)) OR (scanbod*)) OR (dental impression*)) OR (implant impression*)

O: (((((((((((((((((((((((patient satisfaction[MeSH Terms]) OR (outcome, patient reported[MeSH Terms])) OR (patient reported outcome[MeSH Terms])) OR (patient reported outcome measure[MeSH Terms])) OR (patient reported outcome measures[MeSH Terms])) OR (patient reported outcomes[MeSH Terms])) OR (nausea[MeSH Terms])) OR (patient preference[MeSH Terms])) OR (patient preferences[MeSH Terms])) OR (patient comfort[MeSH Terms])) OR (assessment, patient outcome[MeSH Terms])) OR (patient outcome assessment[MeSH Terms])) OR (patient perception*)) OR (patient acceptance)) OR (patient anxiety)) OR (patient discomfort)) OR (patient satisfaction)) OR (patient reported outcome*)) OR (PROs)) OR (PROMs)) OR (nausea)) OR (patient preference*)) OR (patient comfort)) OR (taste)) OR (gag reflex)

Search strategy for Embase:

('dental implant'/exp OR 'implant supported dental prosthesis'/exp OR 'single tooth dental implant'/exp OR 'endosseous dental implantation'/exp

OR 'dental implant*' OR 'implant crown*' OR 'implant bridge*' OR 'implant FDP' OR 'implant fixed partial denture*'

OR 'implant supported single unit crown*' OR 'fixed implant restoration*' OR 'fixed implant prosthesis' OR 'fixed implant prostheses'

OR 'all on four' OR 'all-on-4' OR 'all on six' OR 'all-on-6' OR 'full arch fixed denture*'

OR 'screw retained' OR 'cement retained' OR 'full arch restoration' OR 'implant restoration*' OR 'single unit dental implant*')

AND

('dental impression technique'/exp OR 'dental impression material'/exp OR 'computer aided design'/exp

OR 'digital impression*' OR 'dental impression*' OR 'intraoral scan*' OR 'optical impression*'

OR 'digital scan*' OR 'dental scanner*' OR 'scanbod*' OR 'implant impression*')

AND

('patient satisfaction'/exp OR 'patient reported outcome'/exp OR 'patient preference'/exp OR 'nausea'/exp

OR 'taste'/exp OR 'anxiety'/exp OR 'discomfort'/exp OR 'patient outcome assessment'/exp

OR 'patient reported outcome*' OR 'patient preference*' OR 'patient comfort'

OR 'patient perception*' OR 'patient acceptance' OR 'patient anxiety'

OR 'patient discomfort' OR 'patient satisfaction' OR 'PROs' OR 'PROMs'

OR 'gag reflex')

Search strategy for Scopus:

(TITLE-ABS-KEY("dental implant*" OR "implant crown*" OR "implant bridge*" OR "implant FDP" OR "implant fixed partial denture*"

OR "implant supported single unit crown*" OR "fixed implant restoration*" OR "fixed implant prosthesis" OR "fixed implant prostheses"

OR "all on four" OR "all-on-4" OR "all on six" OR "all-on-6" OR "full arch fixed denture*" OR "screw retained" OR "cement retained"

OR "full arch restoration" OR "implant restoration*" OR "single unit dental implant*"))

AND

(TITLE-ABS-KEY("dental impression*" OR "digital impression*" OR "intraoral scan*" OR "optical impression*" OR "digital scan*"

OR "dental scanner*" OR "scanbod*" OR "implant impression*" OR "CAD/CAM" OR "computer aided design"))

AND

(TITLE-ABS-KEY("patient satisfaction" OR "patient reported outcome*" OR "patient preference*" OR "patient comfort" OR "patient perception*" OR "patient acceptance" OR "patient anxiety" OR "patient discomfort" OR "PROs" OR "PROMs" OR "taste" OR "gag reflex" OR "nausea"))

Search strategy for Cochrane Library:

(dental implant* OR implant crown* OR implant bridge* OR implant FDP OR implant fixed partial denture* OR implant supported single unit crown* OR fixed implant restoration* OR fixed implant prosthesis OR fixed implant prostheses OR all on four OR all on six OR full arch fixed denture* OR screw retained OR cement retained OR full arch restoration OR implant restoration* OR single unit dental implant*) AND (dental impression* OR digital impression* OR intraoral scan* OR optical impression* OR digital scan* OR dental scanner* OR scanbod* OR implant impression* OR CAD/CAM) AND (patient satisfaction OR patient reported outcome* OR patient preference* OR patient comfort OR patient perception* OR patient acceptance OR patient anxiety OR patient discomfort OR PROs OR PROMs OR gag reflex OR nausea OR taste)

Supplementary Table S1: Main Table

Authors	Year	Number of patients	Number of implants	Follow-up	Type of restoration	Implant manufacturer	Scan level	Conventional impression material	Intraoral scanner used	Key findings
Beck et al.	2024	27	27	4 years	Single crowns	Straumann, Institut Straumann AG, Basel, Switzerland	Direct to implant connection	Polyether	TRIOS; 3Shape A/S, Copenhagen, Denmark	Patients showed no clear preference for an impression technique but were more likely to recommend the digital method
Carneiro Pereira et al.	2022	34	61	NA	Full-arch restoration	Neodent; Straumann, Curitiba, Brazil	Via transmucosal abutment	Polyvinylsiloxane	TRIOS; 3Shape A/S, Copenhagen, Denmark	A higher preference was stated for digital impressions.
Chen et al.	2024	82	82	NR	Single crowns	Straumann, Institut Straumann AG, Basel, Switzerland	Direct to implant connection	Polyvinylsiloxane	iTero Element; Align Technology Inc	Digital impressions were considered more comfortable by patients.
Corsalini et al.	2024	60	72	1 year	Single crowns	Nobel Biocare, Zurich, Switzerland	Direct to implant connection	Polyvinylsiloxane	TRIOS 3; 3Shape A/S, Copenhagen, Denmark	Patients rated higher the digital workflow.
De Angelis et al.	2020	122	170	NR	Single crowns, 3-unit FDPs	Straumann, Institut Straumann AG, Basel, Switzerland	Direct to implant connection	Polyether	CEREC AC Omnicam (Sirona Dental System, Long Island City, N.Y., U.S.A.)	All patients would prefer the digital workflow for future implant restorations.

De Angelis N et al.	2023	150	600	2 years	Full-arch restoration	Straumann, Institut Straumann AG, Basel, Switzerland	Via transmucosal abutment	Polyvinylsiloxane	TRIOS 3; 3Shape A/S, Copenhagen, Denmark	Patients were equally satisfied.
Delize et al.	2019	31	31	NR	Single crowns	MIS Implants Technologies Ltd., Dentsply Sirona	Direct to implant connection	Polyvinylsiloxane	TRIOS 2; 3Shape A/S, Copenhagen, Denmark	Higher patient satisfaction and convenience was achieved using the digital technique.
Di Fiore et al.	2018	10	10	NR	Single crowns	NR	Direct to implant connection	Polyether	CerEC AC Omnicam (Sirona Dental System, Long Island City, N.Y., U.S.A.)	Higher patient comfort achieved with the digital impression.
Gintaute et al.	2023	20	40	NR	3-unit FDPs	Straumann, Institut Straumann AG, Basel, Switzerland	Direct to implant connection	Polyether	TRIOS 3; 3Shape A/S, Copenhagen, Denmark or Virtuo Vivo/ Dental Wings Inc., Montreal, Canada	Patients favoured the digital impression in terms of satisfaction.
Guo et al.	2019	20	20	NR	Single crowns	Straumann, Institut Straumann AG, Basel, Switzerland	Direct to implant connection	Polyether	TRIOS 1; 3Shape A/S, Copenhagen, Denmark	The immediate digital impression presented higher patient satisfaction.
Joda et al.	2018	40	40	3 years	Single crowns	Straumann, Institut Straumann AG, Basel, Switzerland	Direct to implant connection	Polyether	iTERO	PROMs of posterior implant crowns manufactured in complete digital and hybrid workflows revealed comparable high levels of satisfaction on the overall treatment outcome.

Kunavisarut et al.	2022	40	40	1 week after	Single crowns	Straumann, Institut Straumann AG, Basel, Switzerland	Direct to implant connection	Polyether	TRIOS; 3Shape A/S, Copenhagen, Denmark	Patient-reported outcomes showed higher results when the impression was digital.
Lee et al.	2022	30	30	NR	Single crowns	Straumann, Institut Straumann AG, Basel, Switzerland	Direct to implant connection	Polyvinylsiloxane	iTero Element; Align Technology Inc	The digital impressions resulted in better results concerning patient-reported outcomes and efficiency.
Mangano et al.	2018	50	50	1 year	Single crowns	Exacone, Leone Implants, Sesto Fiorentino, Italy	Direct to implant connection	Polyvinylsiloxane	CS 3600, Carestream Dental, Rochester, NY, USA	Patients preferred the digital workflow.
Schepke et al.	2015	100	50	NR	Single crowns	Astra Tech Implant System, Dentsply Sirona, Sweden	Direct to implant connection	Polyether	Cerec Omnicam; Sirona	The digital impression procedure reported less inconvenience, less fear of repeating the impression and helplessness during the procedure and higher patient satisfaction.

Seth et al.	2024	40	40	NR	Single crowns	Astra Tech Implant System, Dentsply Sirona, Sweden	Direct to implant connection	Polyether	CEREC Omnicam; Dentsply Sirona	The digital impression technique was mostly preferred (80%) compared to the conventional technique (2%), while 18% reported no preference.
Wismeijer	2014	60	41	NR	Single crowns, FDPs	Straumann, Institut Straumann AG, Basel, Switzerland	Direct to implant connection	Polyether	iTero; Align Technology Inc	The patient preference was statistically significant higher for the digital impression.
Vavrickova et al.	2024	45	31	NA	Single crowns	NR	NR	Polyvinylsiloxane	TRIOS Move+; 3Shape A/S, Copenhagen, Denmark	Digital impressions were considered preferrable by the patients. However, discomfort or difficulties during this negatively affected the patients' attitude to it.

Supplementary Table S2: Summary of the Quality Assessment

Authors	Year of publication	Type of study	Tool	RoB assessment
Beck et al.	2024	RCT	RoB 2.0	Some concerns
Carneiro Pereira et al.	2022	Cross-sectional	NIH tool for cohort and cross-sectional studies	Low
Chen et al.	2024	Self-controlled case study	NIH Quality-Assessment Tool for Before–After (No Control) Studies	Moderate
Corsalini et al.	2024	RCT	RoB 2.0	High
De Angelis et al.	2020	Retrospective	NIH tool for cohort and cross-sectional studies	Moderate

De Angelis N et al.	2023	Retrospective	NIH tool for cohort and cross-sectional studies	Moderate
Delize et al.	2019	non-randomised, crossover clinical trial	JBI Critical Appraisal Checklist for Quasi-Experimental Studies	Moderate
Di Fiore et al.	2018	non-randomised, crossover clinical trial	JBI Critical Appraisal Checklist for Quasi-Experimental Studies	Moderate
Gintaute et al.	2023	crossover RCT	RoB 2.0 for crossover	Low
Guo et al.	2019	non-randomised clinical trial	JBI Critical Appraisal Checklist for Quasi-Experimental Studies	Moderate
Joda et al.	2018	crossover RCT	RoB 2.0 for crossover	Low

Kunavisarut et al.	2022	RCT	RoB 2.0	Some concerns
Lee et al.	2022	crossover RCT	RoB 2.0 for crossover	Some concerns
Mangano et al.	2018	RCT	RoB 2.0	Some concerns
Schepke et al.	2015	non-randomised clinical trial	JBI Critical Appraisal Checklist for Quasi-Experimental Studies	Moderate
Seth et al.	2024	crossover RCT	RoB 2.0 for crossover	Some concerns
Wismeijer	2014	crossover RCT	RoB 2.0 for crossover	High
Vavrickova et al.	2024	Cross-sectional	NIH tool for cohort and cross-sectional studies	Moderate
